# Resuscitation in Oncology: Limits, Ethics, Practice, and Humanity

**DOI:** 10.3390/curroncol33040202

**Published:** 2026-03-31

**Authors:** Lea Andjelković, Milan Krnojelac, Iztok Potočnik

**Affiliations:** 1 Department of Anesthesiology Intensive Therapy, University Medical Centre Ljubljana, 1000 Ljubljana, Slovenia; 2 Division of Internal Medicine, University Medical Centre Ljubljana, 1000 Ljubljana, Slovenia; milan.krnojelac@kclj.si; 3 Department of Anaesthesiology and Intensive Care, Institute of Oncology, 1000 Ljubljana, Slovenia; ipotocnik@onko-i.si

**Keywords:** resuscitation, oncology, cancer, ethics, palliative care, DNR, intensive care, burnout, debriefing, dignity, end-of-life care, medical responsibility

## Abstract

Introduction: Cardiopulmonary resuscitation (CPR) is one of the most consequential decisions in clinical medicine—a pivotal moment between life and death where science, ethics, and humanity intersect. Although advances in systems of care, technology, and training have refined technique and logistics, outcomes do not consistently result in meaningful, neurologically intact survival. In oncology—where disease trajectories are heterogeneous, treatment burdens substantial, and organ reserve often limited—these tensions are especially pronounced. Methods and approaches: This manuscript examines resuscitation as a medical, ethical, and human process, with explicit focus on patients with cancer. We review contemporary strategies for early recognition of deterioration (MEWS, NEWS, MET activation), team preparedness through Immediate Life Support (ILS), and structured decision-making at the boundaries of resuscitation. We also address communication with patients and families, the legal framework of Do-Not-Resuscitate (DNR) orders, and the distinctions among treatment forgoing, palliative sedation, and euthanasia, emphasising oncology-specific considerations such as metastatic burden, treatment intent (curative vs. palliative), performance status, and organ reserve. Results and discussion: The overall effectiveness of resuscitation remains modest (approximately 5–20% survival), highlighting the importance of prevention and early intervention. In cancer care, the limits of resuscitation are both clinical and ethical, requiring proportionality between the likely benefit and the risks of prolonging suffering, careful attention to prognosis and expected neurological outcomes, and rigorous alignment with goals of care. Early and ongoing involvement of palliative services, along with robust long-term care pathways, provides humane, value-concordant alternatives for patients with advanced disease. Psychotherapists and chaplains play integral roles in supporting families and clinical staff. Structured post-event debriefing and system-level safeguards are essential to mitigate burnout and moral distress within oncology teams. Initiating or discontinuing resuscitation in oncology requires expertise, empathy, and moral clarity. Dignity-preserving care depends on aligning interventions with patient values and realistic clinical endpoints. Acceptance of the natural course of dying represents an important component of responsible and patient-centred medical care.

## Simple Summary

Resuscitation is often considered a life-saving intervention, but in patients with cancer it presents complex medical and ethical challenges. Although advances in techniques and training have improved emergency responses, survival after cardiac arrest remains low, particularly in those with advanced cancer. This paper discusses when resuscitation may be beneficial and when it may only prolong suffering. It highlights the importance of early recognition of clinical deterioration, clear communication with patients and families, and timely discussions about care goals, including do-not-resuscitate decisions. The authors emphasise that palliative care and ongoing support are not signs of treatment failure, but rather serve to preserve dignity and quality of life. They also consider the emotional impact on healthcare teams and the need for structured support. These insights can inform future research, improve clinical practice, and shape policies on end-of-life care in oncology.

## 1. Introduction

Resuscitation after cardiac arrest is one of the most critical and ethically complex moments in medicine. Although advances in cardiopulmonary resuscitation (CPR) and the implementation of the “chain of survival” have improved outcomes, decisions about when to initiate, continue, or terminate resuscitation remain challenging. Contemporary guidelines emphasise coordinated systems of care, early defibrillation, and effective team response, while recognising that technical excellence must be combined with ethical judgement and patient-centred decision-making [1,2].

These situations raise fundamental ethical questions about balancing potential benefit against the risk of prolonging suffering. Current guidance highlights the importance of aligning resuscitation efforts with patient values, prognosis, and the proportionality of treatment [3]. The updated European Resuscitation Council (ERC) Guidelines (2025) further emphasise ethical decision-making in resuscitation, including respect for patient autonomy and structured processes for withholding or terminating resuscitation when recovery is unlikely [4].

In oncology, resuscitation decisions are particularly complex due to heterogeneous prognoses, treatment intent, and variable physiological reserve. Survival after in-hospital CPR in patients with cancer ranges from approximately 12% to 31%, with considerably poorer outcomes in those with advanced or metastatic disease. These realities highlight the importance of careful clinical judgement, early advance care planning, and integration of palliative care principles when considering resuscitation in patients with cancer. Reported outcomes of CPR in patients with cancer vary considerably depending on disease stage and clinical setting. While smaller cohort studies report survival to hospital discharge between 15% and 31%, large population-based datasets of patients with advanced or metastatic cancer demonstrate mortality rates exceeding 80% (Table 1) [5,6,7,8,9,10].

### Literature Selection Approach

This narrative review is based on a structured search of the medical literature. Relevant publications were identified through searches of PubMed databases from 2000 to 2025 using combinations of the following keywords: cardiopulmonary resuscitation, cancer, oncology, do-not-resuscitate, intensive care, palliative care, end-of-life care, medical emergency team, early warning scores, and resuscitation ethics. Priority was given to international guidelines, consensus statements, and systematic reviews from major professional societies, including the European Resuscitation Council (ERC), the American Heart Association (AHA), and the European Society of Intensive Care Medicine (ESICM). Publications were selected for their relevance to resuscitation decision-making in patients with cancer, ethical considerations, and the integration of acute and palliative care principles.

## 2. Defining Resuscitation: Concepts, Scope, and Clinical Objectives (Revised)

Resuscitation (Latin reanimatio—“reviving”) refers to a coordinated set of time-critical interventions aimed at restoring circulation and/or ventilation and maintaining organ perfusion until definitive treatment is provided. Contemporary practice distinguishes two complementary levels of care. Basic life support (BLS) includes high-quality chest compressions with minimal interruptions, rescue breathing when indicated, and early use of an automated external defibrillator (AED). BLS can be initiated by trained laypersons or first responders and is crucial because the first minutes after cardiac arrest are often decisive for survival and neurological outcome [1,2].

Advanced life support (ALS) builds on BLS and includes advanced airway management, manual defibrillation for shockable rhythms, rhythm-guided pharmacotherapy, and structured algorithms for identifying and treating reversible causes of cardiac arrest. These interventions are followed by organised post-cardiac arrest care in the intensive care unit [1,2,11].

According to the 2025 European Resuscitation Council (ERC) Guidelines, resuscitation should be understood within a broader systems-of-care framework that integrates early recognition of cardiac arrest, rapid activation of emergency response systems, effective team performance, and coordinated post-cardiac arrest management. The immediate goal of resuscitation is the return of spontaneous circulation with adequate oxygen delivery, while the ultimate objective is survival with acceptable neurological function and quality of life. Because successful resuscitation does not always lead to meaningful recovery, the ERC guidelines emphasise that resuscitation decisions must also consider prognosis, proportionality of treatment, and patient values [4].

## 3. Resuscitation Effectiveness: Current Outcomes and Opportunities for Improvement

Despite advances in technology, increased availability of automated external defibrillators, and improved training of healthcare professionals, survival after cardiac arrest remains limited. Contemporary reports consistently show modest survival rates and significant neurological morbidity, highlighting variability across healthcare systems and the ongoing need to improve resuscitation practice [1,2,3,11].

The 2025 ERC Guidelines emphasise that improving outcomes requires optimisation of the entire chain of survival. This includes early recognition of clinical deterioration, timely activation of emergency response systems, high-quality cardiopulmonary resuscitation with minimal interruptions, early defibrillation for shockable rhythms, and structured post–cardiac arrest care. Continuous monitoring, early identification of warning signs, and rapid response team activation may prevent cardiac arrest or improve outcomes when arrest occurs [4].

Effective resuscitation also depends on provider competence, coordinated team dynamics, and systematic performance evaluation within learning healthcare systems. For this reason, the ERC highlights the importance of structured training programmes such as Immediate Life Support (ILS) and Advanced Life Support (ALS), which aim to improve the early response to cardiac arrest and strengthen team-based resuscitation performance [4].

Importantly, the ERC guidelines also emphasise that resuscitation should be considered an ethically proportionate intervention. Clinicians must assess whether attempted resuscitation is likely to provide meaningful benefit or merely prolong the dying process. Such decisions require integration of clinical prognosis, patient values, and goals of care within responsible and patient-centred medical practice [4].

## 4. Early Recognition of Deterioration: The Most Effective Route to Better Outcomes

**Figure 1:**
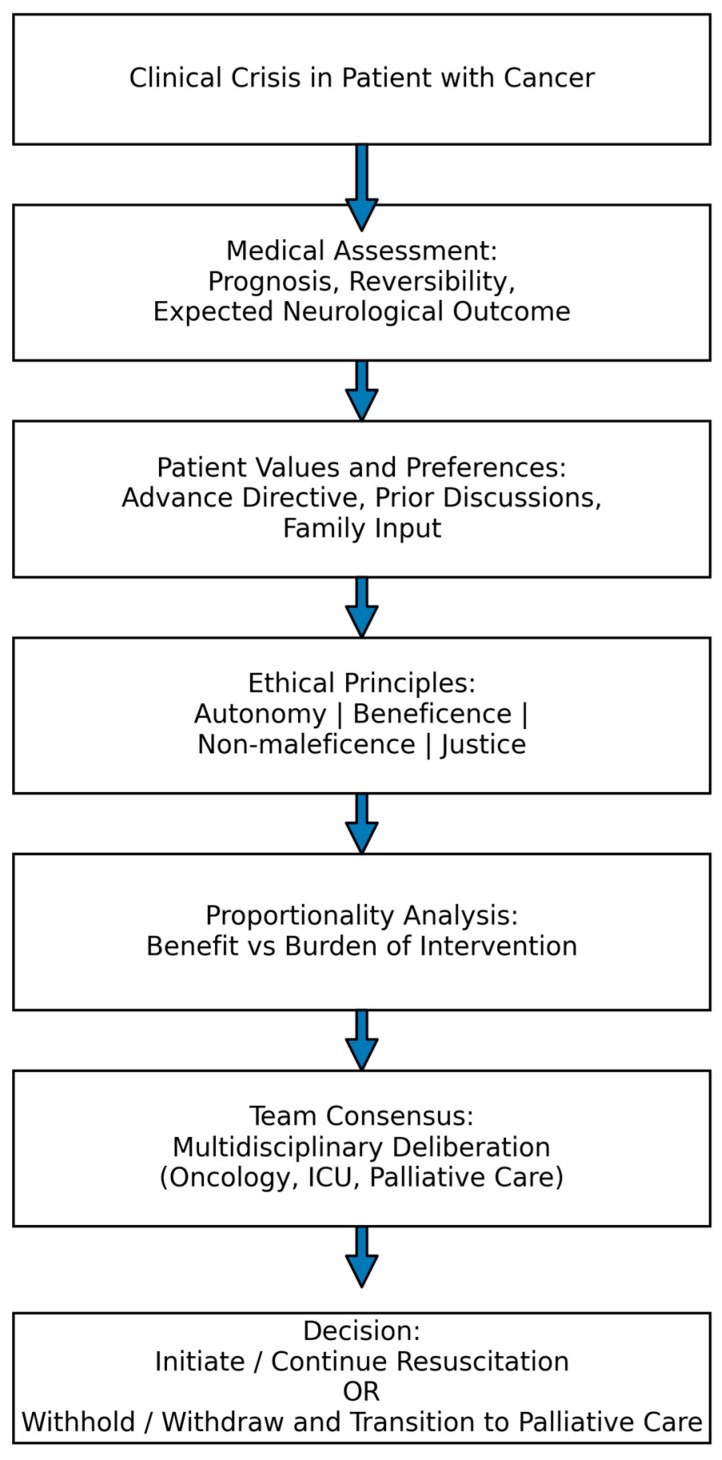
Ethical decision schema.

Effective resuscitation begins well before cardiac arrest. Timely identification of clinical decline—evidenced by changes in consciousness, respiratory pattern, blood pressure, pulse, or oxygen saturation—is critical for prevention and optimising outcomes. To support this vigilance, standardised Early Warning Scores (EWS) provide objective, vital sign–based risk stratification at the bedside. The two most widely used systems are the Modified Early Warning Score (MEWS) and the National Early Warning Score (NEWS). Both rely on simple, reproducible measurements to identify patients whose condition is deteriorating—even before an overt critical event. MEWS, one of the earliest frameworks, integrates five physiological variables (heart rate, systolic blood pressure, respiratory rate, body temperature, and level of consciousness per AVPU), with each parameter weighted by its deviation from normal; the total score defines the risk tier, with higher scores indicating a greater likelihood of acute deterioration and the need for escalation [1,2,3,11]. NEWS, developed by the Royal College of Physicians and now widely used across Europe, builds on this approach by including oxygen saturation (SpO_2_), the use of supplemental oxygen, and, in some versions, urine output. By capturing respiratory compromise more precisely, NEWS improves risk estimation in patients with pulmonary disease and in oncology populations after major surgery. Routine graphical charting of NEWS or MEWS enhances trend recognition, standardises communication, and supports structured decision-making during handovers and escalation [11,12]. When prespecified thresholds are reached, activation of a Medical Emergency Team (MET) enables rapid expert assessment and targeted intervention, often averting cardiac arrest and demonstrably reducing mortality and the subsequent need for resuscitation. Accordingly, MEWS/NEWS and MET activation protocols are integral to safe inpatient care and are key links in the chain of survival [1,2,12]. In line with this approach, many hospitals are moving from traditional “code” teams to MET models—multidisciplinary groups mobilised at the onset of deterioration rather than at arrest—thus bridging the ward and the ICU, enabling timely intervention, preventing arrests, and improving survival (Figure 1). Early action yields multiple benefits: fewer resuscitations, better neurological and functional outcomes after successful resuscitation, and reduced operational burden on emergency services and ward staff. In short, the most effective resuscitation is the one that is prevented. These principles are especially important in oncology, where proactive surveillance and rapid escalation can prevent decompensation in patients whose physiological reserve may be compromised by malignancy or treatment [1,2,8,11,12].

However, the use of generic Early Warning Scores (EWS) in oncology populations presents unique challenges that limit their discriminatory value. Oncology patients often have altered baseline physiology due to malignancy, treatment-related immunosuppression, and chemotherapy-induced cytopenias, which may render standard vital sign thresholds less predictive [13]. In a retrospective analysis of 840 patients at an oncology hospital in the North West of England, only respiratory rate and temperature showed statistically significant predictive power for clinical deterioration. Blood pressure and pulse rate did not have statistically significant predictive value. The study concluded that the Modified Early Warning Score (MEWS) had poor value in predicting critical care admission and 30-day mortality [14]. In a systematic review of seven studies, only two included prospective validation of EWS in patients with cancer, while the other five were retrospective. Lower reliability was also attributed to small sample sizes, single-institution analyses, and the retrospective nature of the studies [13]. A large retrospective cohort study involving 19,739 hospitalised cancer patients developed a deep learning-based Early Warning Score specifically for oncology populations (Can-EWS). By incorporating absolute vital sign values and changes between consecutive measurements, the model demonstrated significantly improved predictive performance compared with traditional scores such as MEWS (AUROC 0.946 vs. 0.589). Importantly, the model also reduced false alarm rates while maintaining high sensitivity for predicting clinical deterioration, defined as in-hospital cardiac arrest or unexpected ICU transfer [15].

## 5. Defining the Limits of Resuscitation in Cancer Care

The limits of resuscitation are defined by medical, ethical, and legal considerations. Medically, these limits are reached when injuries are incompatible with life or when irreversible multi-organ failure is present. In patients who are actively dying, attempted resuscitation is generally considered futile and may increase suffering. The 2025 European Resuscitation Council (ERC) Guidelines emphasise that decisions about initiating or withholding resuscitation should be based on careful clinical assessment, prognosis, and the likelihood of achieving a meaningful recovery [4].

Ethically, the central question is whether an intervention provides net benefit while respecting the patient’s dignity and previously expressed wishes. Attempting resuscitation without a realistic prospect of recovery may expose patients to non-beneficial interventions and additional suffering. Contemporary guidance therefore emphasises early and proactive communication with patients and, when appropriate, with family members or surrogate decision-makers. Shared decision-making and advance care planning are key elements of responsible resuscitation practice, allowing clinicians to align medical interventions with the patient’s care-based goals and values [16,17].

Legally, these limits are commonly implemented through instruments such as do-not-resuscitate (DNR) orders and advance directives. A DNR order does not represent abandonment of care but rather protects patients from interventions unlikely to restore a meaningful health state. The ERC guidelines highlight the importance of clear documentation, transparency in decision-making, and institutional policies that support clinicians in applying consistent and ethically justified limitations of treatment [16,18].

These determinations are particularly complex in oncology, where prognosis may vary widely and therapeutic responses can sometimes be unpredictable. Decisions must therefore be individualised, taking into account disease stage, performance status, treatment intent, and the patient’s preferences. When resuscitation would be non-beneficial and unlikely to improve survival or quality of life, clinicians should provide leadership in redirecting care towards comfort-focused and palliative approaches.

## 6. Resuscitation at the End of Cancer Care: Ethical Dilemmas and Decisions

Determining whether to initiate or discontinue resuscitation is among the most challenging decisions in clinical practice. Physicians often navigate complex tensions between professional responsibility, ethical obligations, and compassion for patients and families. Requests to “do everything” frequently reflect fear, hope, and emotional distress; however, such requests may conflict with clinical realities and the ethical obligation to avoid non-beneficial interventions [16,17].

Ethical decision-making in resuscitation is commonly framed by four core principles of biomedical ethics: autonomy, beneficence, non-maleficence, and justice. Respect for autonomy requires consideration of the patient’s values, previously expressed preferences, and goals of care. Beneficence and non-maleficence guide clinicians towards interventions that provide meaningful benefit while avoiding treatments that merely prolong suffering. Justice relates to the responsible and equitable allocation of healthcare resources.

The 2025 ERC Guidelines emphasise that decisions regarding the initiation, continuation, or termination of resuscitation should be embedded within a broader framework of shared decision-making and advance care planning. Ethical evaluation is therefore not a single moment but an ongoing process involving communication with the patient, family members or surrogates, and the interdisciplinary healthcare team. In certain circumstances, professional responsibility requires recognising when further resuscitative attempts are unlikely to achieve meaningful recovery and when a transition towards comfort-focused care is more consistent with the patient’s goals and dignity [4,16,17].

## 7. Palliative and Long-Term Care in Cancer Patients

Palliative care has historically developed within oncology and has become an essential part of comprehensive cancer care. In Slovenia, palliative services have been established for several decades, initially at the Institute of Oncology Ljubljana and later expanding to other hospitals and regional healthcare institutions. Mobile palliative care teams operate in several centres and provide multidisciplinary support to patients with advanced illness, a substantial proportion of whom have cancer.

Palliative care does not signify abandonment of treatment but rather a shift in therapeutic goals from cure to optimising quality of life (10–12). Its core principles include relief of pain and other distressing symptoms, attention to psychological, social, and spiritual needs, and support for patients and their families throughout the course of serious illness. The ERC 2025 Guidelines emphasise that early integration of palliative care is compatible with high-quality resuscitation practice, particularly when the likelihood of meaningful recovery after cardiac arrest is limited [19,20,21].

Long-term care complements palliative care by supporting individuals with chronic and progressive conditions such as advanced cancer, heart failure, or severe frailty. When effectively organised, these services enable care in home or community settings, reduce avoidable hospital admissions, and improve continuity and safety of care.

In the context of resuscitation decision-making, palliative and long-term care provide essential alternatives to disproportionate medical escalation. Early involvement of palliative care specialists facilitates timely discussions about goals of care, improves alignment between treatments and patient preferences, and reduces the likelihood of non-beneficial interventions at the end of life. By integrating curative, supportive, and palliative approaches, clinicians can ensure that care remains consistent with prognosis, proportionality, and the patient’s values [19,20,21,22,23,24].

## 8. Clinical Decision-Making, Goals of Care, and Code Status in Oncology

Forgoing treatment refers to the decision not to initiate or to discontinue medical interventions that would only prolong suffering without providing meaningful benefit to the patient. This approach is grounded in respect for the natural course of disease, the patient’s wishes, and the preservation of human dignity [16,17,22].

Two operational modalities are commonly distinguished. Withholding refers to the deliberate decision not to start a proposed intervention when it is considered futile or disproportionately burdensome, such as not initiating mechanical ventilation or cardiopulmonary resuscitation in a terminally ill patient. Withdrawing refers to the decision to discontinue an intervention already in progress once it becomes clear that it no longer provides benefit, for example, stopping ventilatory support or artificial nutrition in end-stage disease. Although the distinction may appear largely semantic, it carries ethical significance: withholding means that treatment is never initiated, whereas withdrawing involves stopping treatment once it has become non-beneficial. In both cases, the intention is not to cause death but to avoid prolonging suffering and to respect the patient’s dignity [16,25].

These practices must be clearly distinguished from euthanasia, which involves an active intervention intended to cause death, typically through the administration of medication that leads to rapid and peaceful dying. In Slovenia, euthanasia is prohibited under criminal law, whereas several countries, including the Netherlands, Belgium, and Canada, permit it under strictly regulated legal conditions that generally require an explicit and repeatedly confirmed patient request and the presence of unbearable and incurable suffering [22,23,25].

Similarly, physician-assisted suicide (PAS) differs from euthanasia in that the clinician does not perform the life-ending act but provides the means that the patient uses independently to end their life. PAS is legally permitted in some jurisdictions, such as Switzerland, the US state of Oregon, and Canada, under strict regulatory frameworks requiring repeated voluntary requests and confirmation of decision-making capacity [20,23,25]. In Slovenia and most European countries, however, PAS remains prohibited.

In contrast, forgoing treatment does not cause death but allows the underlying disease to follow its natural course. This approach is consistent with the ethical principle of non-maleficence (primum non nocere) and with the patient’s right to decline interventions that do not provide meaningful benefit [16,19,22].

Within contemporary palliative medicine, the primary goal is neither to hasten nor to prolong death but to ensure a peaceful, painless, and dignified dying process. An important intervention in this context is palliative sedation, defined as the proportional use of medication to intentionally reduce consciousness in patients experiencing refractory and unbearable suffering that cannot be relieved by other therapeutic measures [22,23,24]. It is reserved for the final phase of life and aims to relieve symptoms such as severe pain, dyspnoea, anxiety, or intractable distress.

When appropriately applied, palliative sedation does not hasten death but alleviates suffering and enables a dignified dying process. It differs fundamentally from euthanasia in both intention and clinical practice: the goal is symptom relief, not the deliberate induction of death [19,20,22]. Ethical and procedural requirements include a clearly documented indication of refractory symptoms, informed consent from the patient or authorised surrogate decision-maker, and a multidisciplinary clinical decision based on proportionality, ongoing reassessment, and respect for the patient’s values and goals of care [19,23,24].

In modern palliative care, palliative sedation is recognised as an ethically acceptable and compassionate intervention when all other avenues of symptom control have been exhausted, ensuring that patients do not experience avoidable suffering at the end of life.

## 9. Family, Psychosocial, and Spiritual Support in Oncologic Resuscitation

Confrontation with dying and death elicits powerful emotional responses in all participants—patients, relatives, and healthcare professionals—ranging from fear and helplessness to anger and guilt. The frequently voiced appeal from families to “do everything” rarely reflects a considered judgement; more often, it expresses despair, uncertainty, and anticipatory grief in the face of loss. The clinical task is to recognise and honour these emotions without judgement, while maintaining therapeutic clarity and compassion [19,21,26,27].

A cornerstone of high-quality care is empathic, structured communication: articulating realistic options, exploring values and preferences, and guiding families from a curative stance towards acceptance when appropriate [21,28]. Within this process, mental health professionals and spiritual care providers are integral. Therapists help bring unspoken experiences—guilt, anger, confusion—to the surface and facilitate the transformation of acute distress into a coherent grieving process. Chaplains and other spiritual companions provide a symbolic and ritual framework—prayer, sacrament, or silent presence—that can lend meaning when language fails [19,21]. Presence and reverent silence often achieve more than extensive explanation.

Deliberate collaboration among physicians, therapists, and clergy creates a psychologically safe environment in which death is understood not as defeat but as part of human wholeness. Such interdisciplinary support benefits not only patients and families but also clinicians who routinely bear intense moral and emotional pressure [21,27,28].

Following every resuscitation—particularly when unsuccessful—a structured team debriefing should be conducted. Debriefing is intended for reflection, learning, and emotional decompression rather than fault-finding; it enables expression of experience, articulation of uncertainty, and consolidation of shared learning. Systematic debriefing preserves team psychological safety, mitigates burnout risk, and supports continuous quality improvement [21,26,27,28]. Routine incorporation of these practices signals a mature professional culture in which excellence encompasses not only technical proficiency but also the emotional and ethical capacity to bear the weight of life and death.

## 10. Criteria for Discontinuing Resuscitation in Patients with Advanced Malignancy

Determining when to terminate resuscitative efforts is among the most challenging judgements in clinical practice, particularly in oncology, where advanced malignancy, treatment-related immunosuppression, and cumulative organ dysfunction narrow the window for meaningful recovery. The clinician must balance evidence-based guidelines and temporal thresholds with the patient’s oncological trajectory and goals of care, exercising fiduciary responsibility amid the moral gravity of the bedside reality. Despite advances in resuscitation science, not every attempt is meaningful in patients with disseminated disease or refractory multi-organ failure, and perseverance is not invariably synonymous with hope. In such contexts, when the likelihood of neurologically intact survival is remote and further intervention would merely prolong the dying process, discontinuation is both ethically sound and professionally justified, provided decisions are aligned with documented preferences (e.g., advance directives or DNR), proportional to prognosis, and consistent with consensus best practice [1,2,16,22].

### Oncology-Specific Factors in Resuscitation Decision-Making

In patients with cancer, decisions about resuscitation should incorporate disease-specific and patient-specific factors that influence the likelihood of meaningful recovery, including oncological prognosis, performance status, treatment trajectory, extent of metastatic disease, organ reserve, comorbidities, and the acute reversibility of the precipitating event. Oncological prognosis considers whether the disease is potentially curable, controlled, or represents progressive metastatic malignancy. Performance status is an important determinant, as poor baseline function (e.g., ECOG ≥ 3) is associated with significantly worse outcomes after cardiac arrest. Treatment trajectory reflects whether the patient is receiving active curative or disease-modifying therapy, or care with purely palliative intent. The extent of metastatic disease, particularly when multiple organs or the central nervous system are involved, may limit the likelihood of neurological recovery. Organ reserve and comorbidities, especially cardiopulmonary, renal, and hepatic function, further influence the potential for recovery. The acute reversibility of the precipitating event is also critical, as conditions such as sepsis during chemotherapy-induced neutropenia may be reversible, whereas progressive multi-organ failure due to advanced malignancy is less likely to benefit from resuscitative efforts.

Considering these parameters helps clinicians apply general resuscitation principles to oncology-specific clinical judgement.

## 11. Evidence-Based Criteria for Discontinuing Resuscitation

According to the European Resuscitation Council (ERC) and the American Heart Association (AHA), termination of resuscitative efforts is appropriate when clearly defined conditions are met [1,2,3,11,12,17]. The updated ERC 2025 guidelines further clarify the ethical and clinical framework for such decisions, emphasising the importance of proportionality, patient preferences, and interdisciplinary consensus. In practice, this applies when persistent cardiac arrest shows no response to correctly executed advanced life support after approximately 20–30 min—despite high-quality chest compressions, defibrillation when indicated, timely administration of medications, and definitive airway management—and when reversible causes have been reasonably assessed and excluded. These reversible causes comprise the “4 Hs and 4 Ts”: hypoxia, hypovolaemia, hypo-/hyperkalaemia (and other profound metabolic derangements), hypothermia; and cardiac tamponade, tension pneumothorax, thromboembolism (e.g., massive pulmonary embolism), and toxicity. Discontinuation is also indicated in the presence of injuries incompatible with life (such as catastrophic disruption of cardiac or cerebral structures, decapitation, or other massive traumatic lesions), or when biological death is confirmed by rigor mortis, post-mortem lividity, or tissue decomposition. A valid treatment-limitation document—most commonly a Do Not Resuscitate (DNR) order or advance directive—likewise mandates that resuscitation not be initiated or be stopped once recognised. Even in the absence of formal documentation, efforts should cease when, in the clinician’s ethical and professional judgement, continuation is not in the patient’s best interest—namely when the expected outcome is prolonged suffering, a vegetative state, or complete device dependence without a realistic prospect of meaningful recovery [16,19,20,22].

When the treating team determines that further efforts are futile, the process should proceed calmly, professionally, and transparently. The team should first recheck all potentially reversible causes (4 Hs and 4 Ts) and reassess for any clinical signs of life, electrocardiographic activity consistent with perfusing rhythms, and end-tidal CO_2_ where available. If, after at least 20–30 min, there are still no signs of circulation, the leader should clearly state the decision—“Stop resuscitation”—so that all team members understand. Compressions are halted, the rhythm is verified, and asystole (or a non-perfusing rhythm without reversibility) is confirmed. The time of death is recorded. The physician then communicates with relatives, explains the clinical course, affirms that all medically indicated measures were undertaken, and creates space for silence, spiritual support, or prayer if desired.

**Figure 2:**
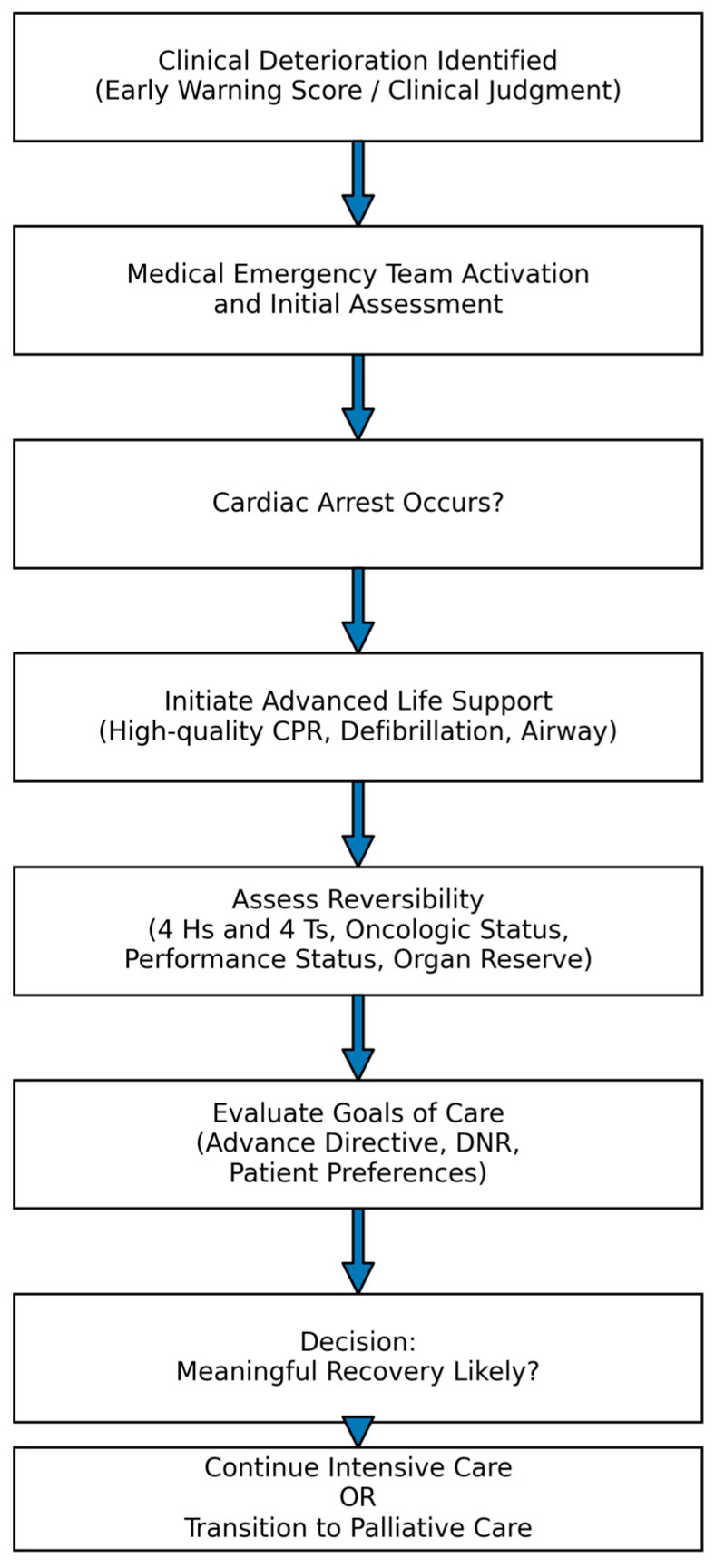
Resuscitation decision flowchart.

Documentation must be precise and complete, including start and end times of resuscitation, interventions performed and the patient’s responses, the rationale for termination, team members present, and a summary of discussions with relatives. Importantly, stopping resuscitation is not abandonment of care; it marks a transition from a curative to a palliative approach, in which the clinician acknowledges the biological limits of life. As ERC guidance underscores, “Discontinuing resuscitation efforts is a clinical and ethical decision, not a failure” [1] (Figure 2).

## 12. Indications for ICU Admission in Oncology

Admission to the intensive care unit (ICU) is among the most demanding decisions in clinical medicine [18,19,22,28]. ICU care involves advanced, often invasive organ-support modalities—mechanical ventilation, vasopressors, renal replacement therapy, invasive haemodynamic monitoring—delivered by a continuously present multidisciplinary team. Such decisions must be based on clear medical indications, a realistic prognosis, and explicit respect for the patient’s values and preferences. The objective of intensive care is not merely to prolong biological existence but to restore recoverable physiology where this remains attainable.

Common indications for ICU admission in this context include acute respiratory failure—such as ARDS, severe pneumonia, or critical upper-airway obstruction—circulatory collapse from shock, malignant arrhythmias, or decompensated heart failure, abrupt impairment of consciousness or coma requiring airway protection and continuous neurological monitoring, acute kidney injury progressing towards multi-organ failure, and systemic crises such as severe sepsis, exsanguinating haemorrhage, polytrauma, or marked postoperative instability. Appropriateness, however, should result from a holistic appraisal of the patient rather than any single laboratory value or physiological parameter, explicitly balancing the probability of meaningful recovery against the risk of merely prolonging suffering [17,18]. In advanced or terminal illness, ICU admission frequently confers no net benefit; at such points it becomes essential to distinguish interventions that plausibly save life from those that only extend the dying process. Clinicians should consider whether a return to an acceptable quality of life is likely, whether proposed interventions align with the patient’s stated wishes, and whether the intensity of treatment would be disproportionate to the underlying prognosis. Where these criteria are not met, redirection of care towards palliation is both professionally and ethically appropriate [19,22].

Within oncology, the boundary between beneficial intensive care and futile persistence is especially delicate. Advanced malignancy commonly involves multi-organ dysfunction, treatment-related immunosuppression, and diminished regenerative capacity; accordingly, the prospects for recovery after prolonged mechanical ventilation or septic shock are often poor [17,19,28,29]. Nevertheless, a cancer diagnosis is not, in itself, a contraindication to ICU care. Decisions should integrate the biological nature of the malignancy (potentially curable versus terminal), the current treatment phase, overall performance status and organ reserve, and—critically—the informed preferences of the patient and family.

The most favourable outcomes occur when complications are reversible and the underlying disease remains controllable (e.g., febrile neutropenia following chemotherapy). By contrast, in terminal, disseminated disease, escalated organ support is frequently ineffective and ethically questionable. Notably, in terminally ill oncology patients, resuscitation success is exceedingly low: after in-hospital cardiac arrest, fewer than 5% of patients with metastatic disease survive, and most survivors sustain serious neurological sequelae [3,29]. Accordingly, ERC (2025) guidance urges advance consideration of resuscitation in such cases and—when the likelihood of recovery is negligible—adoption of a withhold strategy [1,4,22,23].

A cornerstone of ethical practice is a proactive goals-of-care discussion with the patient and family. When the patient retains decision-making capacity, preferences regarding resuscitation should be documented (advance directive). This does not withhold care; it safeguards dignity and the right to a natural death. In situations of uncertainty, decisions should be reached collaboratively—oncologist, anaesthesiologist, internist, palliative-care physician, and, where possible, the patient or family [16,18,22]. Such team-based deliberation favours ethically balanced choices grounded in consensus rather than reactive judgements under duress.

The Belgian/French Societies’ consensus conference underscores that resuscitation in critically ill cancer patients should be guided by individualised assessment of prognosis, reversibility, and patient wishes, with a strong emphasis on multidisciplinary collaboration and early goals-of-care discussions [30].

Admission of an oncology patient to the ICU is never automatic; it requires thoughtful, team-based, ethically grounded judgement. The patient’s benefit must remain central—not merely the technical feasibility of organ support. A clinician who discerns the line between treatment and the prolongation of dying does not extinguish hope; rather, they redirect it towards reality, dignity, and compassion.

## 13. Ethical and Pragmatic Approaches to High-Stakes Clinical Care

Clinical teams working at the intersection of resuscitation and oncology routinely face situations of profound moral, emotional, and cognitive intensity. High-stakes events—whether urgent resuscitation or the progression of a patient’s terminal illness—provoke surges of responsibility, ethical reflection, and stress, demanding rapid, consequential decision-making. Attitudes and behaviours of personnel in these contexts are pivotal: the ability to act decisively while remaining ethically grounded, emotionally present, and collegially supportive determines both patient outcomes and team resilience [19,24,31].

### 13.1. Structured Debriefing and Reflective Practice

Immediate (“hot”) and delayed (“cold”) debriefings after critical events provide essential opportunities for reflection, learning, and emotional processing. Effective debriefings foster a psychologically safe environment in which all team members can speak candidly, hierarchies are temporarily set aside, and mistakes are explored constructively rather than punitively. By articulating emotional responses, reconstructing key decisions, and identifying actionable lessons, teams mitigate feelings of guilt, reduce the risk of burnout, and strengthen trust and cohesion. Embedding debriefing into routine practice exemplifies an ethical commitment to both professional development and humanistic care [24,27,31].

### 13.2. Confronting Mortality with Ethical Clarity

Clinicians in oncology and critical care frequently encounter death and dying, experiences that test moral, emotional, and spiritual capacities. Ethical practice in these contexts requires acknowledging death not as defeat, but as a meaningful event within a patient-centred care trajectory. Decision-making must balance therapeutic intervention with respect for patient autonomy, dignity, and culturally informed values. Structured support—supervision, peer discussion, reflective rounds, and access to psychological or spiritual resources—enables clinicians to navigate these challenges without emotional numbing or moral compromise. Acceptance, communicated clearly and empathetically, often constitutes the most therapeutically valuable response [1,16,22,24].

### 13.3. Burnout, Moral Distress, and Organisational Responsibility

Persistent exposure to life-and-death scenarios can lead to emotional exhaustion, depersonalisation, and diminished professional efficacy. Burnout is compounded by high perceived responsibility, staffing pressures, communication breakdowns, and insufficient institutional support. Prevention and mitigation require multilevel strategies. Organisational strategies include systematic debriefings, protected time for reflection, and psychologically safe communication structures. Team-based approaches involve clearly defined roles, peer-support mechanisms, and reflective rounds to address moral residue. Individual strategies focus on maintaining boundaries between work and personal life, engagement in reflective practice, and attention to physical, emotional, and spiritual well-being.

By combining structured processes, ethical awareness, and a supportive culture, clinical teams can navigate high-stakes scenarios in a manner that promotes sound decision-making, preserves humanistic values, and sustains professional resilience [1,16,22,24,32,33,34].

## 14. Conclusions

Resuscitation in oncology patients must balance medical possibilities with ethical, legal, and human limitations. Optimal care combines technical skill with careful prognostic judgement, goal-concordant planning, and recognition of when interventions no longer offer meaningful recovery. Decisions should be multidisciplinary, patient-centred, and transparently communicated with families, with timely transition to palliative care when appropriate to preserve dignity and minimise suffering.

Supporting clinicians is equally essential. Routine debriefings, peer and psychological support, and organisational structures that protect time, team cohesion, and moral clarity help to mitigate burnout and moral distress, sustaining the capacity for ethical decision-making.

Future directions include developing standardised frameworks for prognostic assessment and decision-making in oncology resuscitation, evaluating structured debriefing and reflective practice for team resilience and patient outcomes, and enhancing multidisciplinary collaboration, patient–family communication, and institutional policies that balance life-sustaining interventions with quality of life.

Ultimately, the most humane care aligns interventions with outcomes that patients value, accompanying them with competence, compassion, and ethical clarity through life’s final stages.

## Figures and Tables

**Table 1:** CPR Outcomes in Cancer Patients—summarizes outcomes of CPR in patients with cancer across several studies and clinical settings. The studies include in-hospital CPR, emergency department resuscitation, code blue events in cancer centres, and out-of-hospital cardiac arrest.

Study	Patient Type/Setting	Number of Patients	Main Outcome
General literature	In-hospital CPR in cancer patients	Various studies	12–31% survive to hospital discharge
Cancer centre study (2024) [5]	Cancer patients receiving CPR in emergency department	100	67% ROSC; 15% survive to discharge
Canadian cancer centre cohort [6]	Code blue in cancer patients	225	31% survive to discharge; 16% alive at 1 year
US Nationwide Inpatient Sample [7]	Hospitalized patients with metastatic cancer receiving CPR	26 070	81.8% mortality
Taiwan Cancer Registry [9]	Stage IV cancer with in-hospital CPR	3 446	82.8% mortality before discharge
French population study [10]	Out-of-hospital cardiac arrest (cancer patients)	207	26.2% hospital survival

## Data Availability

Data sharing is not applicable to this article as no new data were created or analyzed in this study. This article is based on previously published literature.

## References

[1] Berg K.M., Cheng A., Panchal A.R., Topjian A.A., Aziz K., Bhanji F., Bigham B.L., Hirsch K.G., Hoover A.V., Kurz M.C. (2020). Part 7: Systems of Care: 2020 American Heart Association Guidelines for Cardiopulmonary Resuscitation and Emergency Cardiovascular Care. Circulation.

[2] Panchal A.R., Bartos J.A., Cabañas J.G., Donnino M.W., Drennan I.R., Hirsch K.G., Kudenchuk P.J., Kurz M.C., Lavonas E.J., Morley P.T. (2020). Part 3: Adult Basic and Advanced Life Support: 2020 American Heart Association Guidelines for Cardiopulmonary Resuscitation and Emergency Cardiovascular Care. Circulation.

[3] Okubo M., Komukai S., Andersen L.W., Berg R.A., Kurz M.C., Morrison L.J., Callaway C.W. (2024). American Heart Association’s Get with The Guidelines—Resuscitation Investigators Duration of Cardiopulmonary Resuscitation and Outcomes for Adults with In-Hospital Cardiac Arrest: Retrospective Cohort Study. BMJ.

[4] Greif R., Lauridsen K.G., Djärv T., Ek J.E., Monnelly V., Monsieurs K.G., Nikolaou N., Olasveengen T.M., Semeraro F., Spartinou A. (2025). European Resuscitation Council Guidelines 2025 Executive Summary. Resuscitation.

[5] Wechsler A.H., Sandoval M., Viets-Upchurch J., Cruz Carreras M., Page V.D., Elsayem A., Qdaisat A., Yeung S.-C.J. (2024). Prior Advanced Care Planning and Outcomes of Cardiopulmonary Resuscitation in the Emergency Department of a Comprehensive Cancer Center. Cancers.

[6] Yeung S.H.M., Boles R., Munshi L., Moore M., Seedon S., Shah S., Thyagu S., Mehta S. (2023). Resuscitation Outcomes in Patients with Cancer: Experience in a Large Urban Cancer Centre. Can. J. Anaesth..

[7] Malapati S., Singh S.R.K., Kumar R., Hadid T. (2021). Outcomes of In-Hospital Cardiopulmonary Resuscitation for Cardiac Arrest in Adult Patients with Metastatic Solid Cancers: A Nationwide Inpatient Sample Database Analysis from 2012 to 2014. Cancer.

[8] Geelhand de Merxem M., Ameye L., Meert A.-P. (2024). Benefits of Cardiopulmonary Resuscitation in Cancer Patients. Support. Care Cancer.

[9] Lee M.-R., Yu K.-L., Kuo H.-Y., Liu T.-H., Ko J.-C., Tsai J.-S., Wang J.-Y. (2019). Outcome of Stage IV Cancer Patients Receiving In-Hospital Cardiopulmonary Resuscitation: A Population-Based Cohort Study. Sci. Rep..

[10] Weizman O., Eslami A., Bougouin W., Beganton F., Lamhaut L., Jost D., Dumas F., Cariou A., Marijon E., Jouven X. (2024). Sudden Cardiac Arrest in Patients with Cancer in the General Population: Insights from the Paris-SDEC Registry. Heart.

[11] Bray J.E., Grasner J.-T., Nolan J.P., Iwami T., Ong M.E., Finn J., McNally B., Nehme Z., Sasson C., Tijssen J. (2024). Cardiac Arrest and Cardiopulmonary Resuscitation Outcome Reports: 2024 Update of the Utstein Out-of-Hospital Cardiac Arrest Registry Template. Circulation.

[12] Merchant R.M., Topjian A.A., Panchal A.R., Cheng A., Aziz K., Berg K.M., Lavonas E.J., Magid D.J. (2020). Adult Basic and Advanced Life Support, Pediatric Basic and Advanced Life Support, Neonatal Life Support, Resuscitation Education Science, and Systems of Care Writing Groups Part 1: Executive Summary: 2020 American Heart Association Guidelines for Cardiopulmonary Resuscitation and Emergency Cardiovascular Care. Circulation.

[13] Nagarajah S., Krzyzanowska M.K., Murphy T. (2022). Early Warning Scores and Their Application in the Inpatient Oncology Settings. JCO Oncol. Pract..

[14] Cooksley T., Kitlowski E., Haji-Michael P. (2012). Effectiveness of Modified Early Warning Score in Predicting Outcomes in Oncology Patients. QJM.

[15] Ko R.-E., Kim Z., Jeon B., Ji M., Chung C.R., Suh G.Y., Chung M.J., Cho B.H. (2023). Deep Learning-Based Early Warning Score for Predicting Clinical Deterioration in General Ward Cancer Patients. Cancers.

[16] Mentzelopoulos S.D., Slowther A.-M., Fritz Z., Sandroni C., Xanthos T., Callaway C., Perkins G.D., Newgard C., Ischaki E., Greif R. (2018). Ethical Challenges in Resuscitation. Intensive Care Med..

[17] Hirsch K.G., Abella B.S., Amorim E., Bader M.K., Barletta J.F., Berg K., Callaway C.W., Friberg H., Gilmore E.J., Greer D.M. (2024). Critical Care Management of Patients After Cardiac Arrest: A Scientific Statement from the American Heart Association and Neurocritical Care Society. Circulation.

[18] Nates J.L., Nunnally M., Kleinpell R., Blosser S., Goldner J., Birriel B., Fowler C.S., Byrum D., Miles W.S., Bailey H. (2016). ICU Admission, Discharge, and Triage Guidelines: A Framework to Enhance Clinical Operations, Development of Institutional Policies, and Further Research. Crit. Care Med..

[19] Bohula E.A., Landzberg M.J., Menon V., Alviar C.L., Barsness G.W., Crousillat D.R., Jain N., Page R., Wells R., Damluji A.A. (2025). Palliative and End-of-Life Care During Critical Cardiovascular Illness: A Scientific Statement from the American Heart Association. Circulation.

[20] Osinski A., Vreugdenhil G., de Koning J., van der Hoeven J.G. (2017). Do-Not-Resuscitate Orders in Cancer Patients: A Review of Literature. Support. Care Cancer.

[21] Tanaka Gutiez M., Efstathiou N., Innes R., Metaxa V. (2023). End-of-Life Care in the Intensive Care Unit. Anaesthesia.

[22] Kesecioglu J., Rusinova K., Alampi D., Arabi Y.M., Benbenishty J., Benoit D., Boulanger C., Cecconi M., Cox C., van Dam M. (2024). European Society of Intensive Care Medicine Guidelines on End of Life and Palliative Care in the Intensive Care Unit. Intensive Care Med..

[23] Sprung C.L., Truog R.D., Curtis J.R., Joynt G.M., Baras M., Michalsen A., Briegel J., Kesecioglu J., Efferen L., De Robertis E. (2014). Seeking Worldwide Professional Consensus on the Principles of End-of-Life Care for the Critically Ill. The Consensus for Worldwide End-of-Life Practice for Patients in Intensive Care Units (WELPICUS) Study. Am. J. Respir. Crit. Care Med..

[24] Jackson V.A., Emanuel L. (2024). Navigating and Communicating about Serious Illness and End of Life. N. Engl. J. Med..

[25] Bernacki R.E., Block S.D. (2014). American College of Physicians High Value Care Task Force Communication about Serious Illness Care Goals: A Review and Synthesis of Best Practices. JAMA Intern. Med..

[26] Dzeng E., Colaianni A., Roland M., Chander G., Smith T.J., Kelly M.P., Barclay S., Levine D. (2015). Influence of Institutional Culture and Policies on Do-Not-Resuscitate Decision Making at the End of Life. JAMA Intern. Med..

[27] Marik P.E., Zaloga G.P. (2001). CPR in Terminally Ill Patients?. Resuscitation.

[28] Sprung C.L., Ricou B., Hartog C.S., Maia P., Mentzelopoulos S.D., Weiss M., Levin P.D., Galarza L., de la Guardia V., Schefold J.C. (2019). Changes in End-of-Life Practices in European Intensive Care Units From 1999 to 2016. JAMA.

[29] Linebarger J.S., Johnson V., Boss R.D. (2022). Guidance for Pediatric End-of-Life Care. Pediatrics.

[30] Meert A.-P., Wittnebel S., Holbrechts S., Toffart A.-C., Lafitte J.-J., Piagnerelli M., Lemaitre F., Peyrony O., Calvel L., Lemaitre J. (2021). Critically Ill Cancer Patient’s Resuscitation: A Belgian/French Societies’ Consensus Conference. Intensive Care Med..

[31] Arpagaus A., Arpagaus L., Becker C., Gross S., Gössi F., Bissmann B., Zumbrunn S.K., Schuetz P., Leuppi J.D., Aujesky D. (2025). Checklist-Guided Code Status Discussions in Patients for Whom Cardiopulmonary Resuscitation Is Considered Futile: An Analysis of a Randomized Clinical Trial. JAMA Netw. Open.

[32] Milenović M., Matejić B., Vasić V., Frost E., Petrović N., Simić D. (2016). High Rate of Burnout among Anaesthesiologists in Belgrade Teaching Hospitals: Results of a Cross-Sectional Survey. Eur. J. Anaesthesiol..

[33] Banerjee S., Califano R., Corral J., de Azambuja E., De Mattos-Arruda L., Guarneri V., Hutka M., Jordan K., Martinelli E., Mountzios G. (2017). Professional Burnout in European Young Oncologists: Results of the European Society for Medical Oncology (ESMO) Young Oncologists Committee Burnout Survey. Ann. Oncol..

[34] Medisauskaite A., Kamau C. (2017). Prevalence of Oncologists in Distress: Systematic Review and Meta-Analysis. Psychooncology.

